# Factor Xa Inhibitor Suppresses the Release of Phosphorylated HSP27 from Collagen-Stimulated Human Platelets: Inhibition of HSP27 Phosphorylation via p44/p42 MAP Kinase

**DOI:** 10.1371/journal.pone.0149077

**Published:** 2016-02-11

**Authors:** Masanori Tsujimoto, Gen Kuroyanagi, Rie Matsushima-Nishiwaki, Yuko Kito, Yukiko Enomoto, Hiroki Iida, Shinji Ogura, Takanobu Otsuka, Haruhiko Tokuda, Osamu Kozawa, Toru Iwama

**Affiliations:** 1 Department of Neurosurgery, Gifu University Graduate School of Medicine, Gifu, Japan; 2 Department of Pharmacology, Gifu University Graduate School of Medicine, Gifu, Japan; 3 Department of Emergency and Disaster Medicine, Gifu University Graduate School of Medicine, Gifu, Japan; 4 Department of Orthopedic Surgery, Nagoya City University Graduate School of Medical Sciences, Nagoya, Japan; 5 Department of Anesthesiology and Pain Medicine, Gifu University Graduate School of Medicine, Gifu, Japan; 6 Department of Clinical Laboratory, National Center for Geriatrics and Gerontology, Obu, Aichi, Japan; University Hospital Medical Centre, GERMANY

## Abstract

Selective inhibitors of factor Xa (FXa) are widely recognized as useful therapeutic tools for stroke prevention in non-valvular atrial fibrillation or venous thrombosis. Thrombin, which is rapidly generated from pro-thrombin through the activation of factor X to FXa, acts as a potent activator of human platelets. Thus, the reduction of thrombin generation by FXa inhibitor eventually causes a suppressive effect on platelet aggregation. However, little is known whether FXa inhibitors directly affect the function of human platelets. We have previously reported that collagen induces the phosphorylation of heat shock protein 27 (HSP27), a low-molecular weight heat shock protein via Rac-dependent activation of p44/p42 mitogen-activated protein (MAP) kinase in human platelets, eventually resulting in the release of HSP27. In the present study, we investigated the direct effect of FXa inhibitor on the collagen-induced human platelet activation. Rivaroxaban as well as edoxaban significantly reduced the collagen-induced phosphorylation of both HSP27 and p44/p42 MAP kinase without affecting the platelet aggregation. Rivaroxaban significantly inhibited the release of phosphorylated HSP27 from collagen-stimulated platelets but not the secretion of platelet derived growth factor-AB. In patients administrated with rivaroxaban, the collagen-induced levels of phosphorylated HSP27 were markedly diminished after 2 days of administration, which failed to affect the platelet aggregation. These results strongly suggest that FXa inhibitor reduces the collagen-stimulated release of phosphorylated HSP27 from human platelets due to the inhibition of HSP27 phosphorylation via p44/p42 MAP kinase.

## Introduction

Human platelets play pivotal roles in primary haemostasis and repairs of vascular injury [1]. Platelets adhere with the subendothelium via adhesion receptors such as glycoprotein (GP) Ib/IX/V at the first step of thrombus formation [1,2]. GP Ib/IX/V mediate rolling and tethering of platelets by interaction with von Willebrand factor at the injured sites where collagen lies just beneath the endothelium [1,2]. Collagen is well known as a potent coagulant for human platelets via GPVI and integrin α2β1 on the plasma membrane of platelets [3,4]. Activated GPVI causes up-regulation of integrin activity [5], and leads to granule secretion including platelet-derived growth factor (PDGF)-AB [3]. On the other hand, thrombin is rapidly generated from pro-thrombin through the activation of factor X to factor Xa (FXa) on human platelets adhering to injured vessel walls, and leads to the conversion of fibrinogen to fibrin [2,6]. Thrombin is a potent activator of human platelets via specific receptors, protease-activated receptor (PAR)-1 and PAR-4, and plays a crucial role in the initial phase of coagulation cascade [2]. Oral anticoagulants which selectively inhibit FXa are generally recognized as useful therapeutic tools for stroke prevention in non-valvular atrial fibrillation or venous thrombosis [7–9]. Regarding FXa inhibitor-effect on platelet functions, it has been reported that FXa inhibitors reduce the tissue factor-induced platelet aggregation [10,11], whereas the collagen-induced platelet aggregation is hardly affected by FXa inhibitors [12,13]. The reduction of thrombin generation by inhibiting FXa seems to be a possible approach for inhibition of platelet activity. Regarding anticoagulant effects on platelet aggregation, we have previously reported that antithrombin-III (AT-III) reduces the collagen-induced platelet aggregation [14]. However, little is known about the direct effect of FXa inhibitors on platelet function.

Heat shock proteins (HSPs) are expressed in response to a variety of biological stresses such as heat and chemicals [15]. HSPs facilitate the refolding of unfolded proteins or assist in their elimination as molecular chaperones [15]. HSPs have recently been classified into seven families such as HSPA (HSP70), HSPC (HSP90) and HSPB (low-molecular weight HSPs) [16]. HSP27 is one of the members of HSPB with monomeric molecular masses ranging from 15 to 30 kDa. Accumulating evidence suggests that HSP27 has important roles in multiple functions such as stress tolerance, anti-apoptosis, and signal transduction [16–19]. HSP27 induces post-translational modification such as phosphorylation [15,16]. It is currently recognized that human HSP27 is phosphorylated at three serine residues (Ser-15, Ser-78 and Ser-82) [15,20]. HSP27 exists in an unphosphorylated aggregated form under unstimulated condition. Once phosphorylated, HSP27 is rapidly dissociated, resulting in decrease of the size to dimer or monomer [15,21]. The dissociation is necessary for substrate binding and specific functions [21]. It has been reported that collagen stimulates the activation of p38 mitogen-activated protein (MAP) kinase, leading to HSP27 phosphorylation in human platelets [22]. In our previous studies [23,24], we have shown that the collagen-induced phosphorylation of HSP27 via p44/p42 MAP kinase is correlated with platelet granule secretion such as PDGF-AB in human platelets, and that Rac, a low-molecular weight GTP-binding protein, regulates the phosphorylation of HSP27 via p44/p42 MAP. In addition, we have demonstrated that AT-III attenuates the collagen-induced phosphorylation of HSP27 via p44/p42 MAP, resulting in inhibition of PDGF-AB secretion [14]. Furthermore, we have recently reported that phosphorylated HSP27 is released from platelets accompanied with its phosphorylation induced by collagen in type 2 diabetes mellitus (DM) patients [25]. However, the exact role of HSP27 in human platelets remains to be clarified.

In the present study, we investigated the direct effect of FXa inhibitor on the collagen-induced human platelet activation. We herein show that FXa inhibitor suppresses the collagen-induced phosphorylation of HSP27 via p44/p42 MAP kinase, resulting in the inhibition of the phosphorylated HSP27 release.

## Materials and Methods

### Materials

Collagen was purchased from Takeda Austria GmbH (Linz, Austria). Rivaroxaban and edoxaban were kindly provided by Bayer Vital GmbH (Leverkusen, Germany) and Daiichi Sankyo Co., Ltd. (Tokyo, Japan), respectively. Phospho-specific p44/p42 MAP kinase antibodies, p44/p42 MAP kinase antibodies, phospho-specific HSP27 antibodies (Ser-78) and phospho-specific HSP27 antibodies (Ser-82) were purchased from Cell Signaling Technology, Inc. (Beverly, MA). HSP27 antibodies and GAPDH antibodies were obtained from Santa Cruz Biotechnology, Inc. (Santa Cruz, CA). The PDGF-AB enzyme-linked immunosorbent assay (ELISA) kit was obtained from R&D System, Inc. (Minneapolis, MN). The phosphorylated HSP27 ELISA kit was purchased from Enzo Life Science INC. (Farmingdale, NY). ECL Western blotting detection system was obtained from GE Healthcare (Buckinghamshire, UK). Other materials were obtained from commercial sources.

### Subjects

Fifteen healthy volunteers (10 healthy volunteers for rivaroxaban assay and 5 healthy volunteers for edoxaban assay) and 5 patients (4 patients with non-valvular atrial fibrillation and 1 patient with deep venous thrombosis) who have not been taking any antiplatelet medicines, were enrolled in this study (male/female, 1/4; age, 60.0 ± 6.2 years). Written informed consent was obtained from all of the healthy donors and patients after a detailed explanation. The study was approved by the Committee of Ethics of the Gifu University Graduate School of Medicine.

### Preparation of platelets

Blood samples from healthy volunteers were donated and mixed into a 1/10 volume of 3.2% sodium citrate. Blood samples from patients were collected twice; once before administration of rivaroxaban and once after 2 days of daily administration of rivaroxaban, and then mixed into a 1/10 volume of 3.2% sodium citrate. Platelet-rich plasma (PRP) was prepared from blood samples by centrifugation at 155 x g for 12 min at room temperature. Platelet-poor plasma (PPP) was obtained from residual blood by centrifugation at 1,400 x g for 5 min.

### Measurement of platelet aggregation induced by collagen

Platelet aggregation using citrated PRP was carried out in an aggregometer with laser scattering system (PA-200 Kowa Co. Ltd., Tokyo, Japan), which can present the size of platelet aggregates based up particle counting (small size, 9–25 μm; medium size, 25–50 μm; large size, 50–70 μm), as described in detail previously [14,23–25]. Briefly, to examine the effects of FXa inhibitors, PRP from healthy volunteers was pretreated at room temperature with doses of 0, 500 and 1000 ng/ml of rivaroxaban or edoxaban for 15 min. The PRP was then preincubated at 37°C with a stirring speed of 800 rpm for 1 min, and stimulated by collagen. On the other hand, PRP from patients was preincubated at 37°C with a stirring speed of 800 rpm for 1 min followed by the addition of collagen. Platelet aggregation was monitored for 5 min from the beginning of preincubation. The percentage of transmittance of the PRP was recorded as 0%, and that of the appropriate PPP (blank) was recorded as 100%.

After the stimulation with collagen, platelet aggregation was terminated by the addition of ice-cold EDTA (10 mM) solution. The conditional mixture was centrifuged at 10,000 x g at 4°C for 2 min. To measure PDGF-AB and phosphorylated HSP27 as described below, the supernatant was isolated and stored at -20°C. For Western blot analysis, the pellet was washed twice with phosphate-buffered saline, and then lysed and immediately boiled in a lysis buffer containing 62.5 mM Tris/Cl, pH 6.8, 2% sodium dodecyl sulfate (SDS), 50 mM dithiothreitol, and 10% glycerol.

### Western blot analysis

Western blot analysis was performed as described in detail previously [26]. Briefly, SDS-poly-acrylamidegel electrophoresis (PAGE) was performed by the method of Laemmli in a 10% or 12% polyacrylamide gel [27]. The proteins in the gel were transferred onto a polyvinylidene fluoride (PVDF) membrane. The membranes were then blocked with 5% fat-free dry milk in Tris-buffered saline with 0.1% Tween-20 (TBS-T, 20 mM Tris, pH 7.6, 137 mM NaCl, 0.1% Tween) for 2 h before incubation with the indicated primary antibodies.

Peroxidase-labeled anti-mouse IgG or anti-rabbit IgG antibodies were used as the secondary antibodies. The primary and secondary antibodies were diluted to the optimum concentrations with 5% fat-free dry milk in TBS-T. Peroxidase activity on PVDF membrane was visualized on X-ray film by means of an ECL Western blotting detection system according to the manufacturer's protocol.

### Measurement of the levels of PDGF-AB and phosphorylated HSP27

The level of PDGF-AB and phosphorylated HSP27 in the supernatant of the conditional mixture were determined using each ELISA kit according to the manufacturer's instructions.

### Statistical analysis

The data are presented as the means ± SEM. Comparisons of continuous variables were analyzed by using Wilcoxon signed-rank test because of their non-normal distribution. A probability value of <0.05 was considered statistically significant. All statistical analyses were performed using PASW Statistics software, version 18 (SPSS Japan, Tokyo, Japan).

## Results

### Effects of rivaroxaban or edoxaban on the platelet aggregation induced by collagen

**Fig 1:**
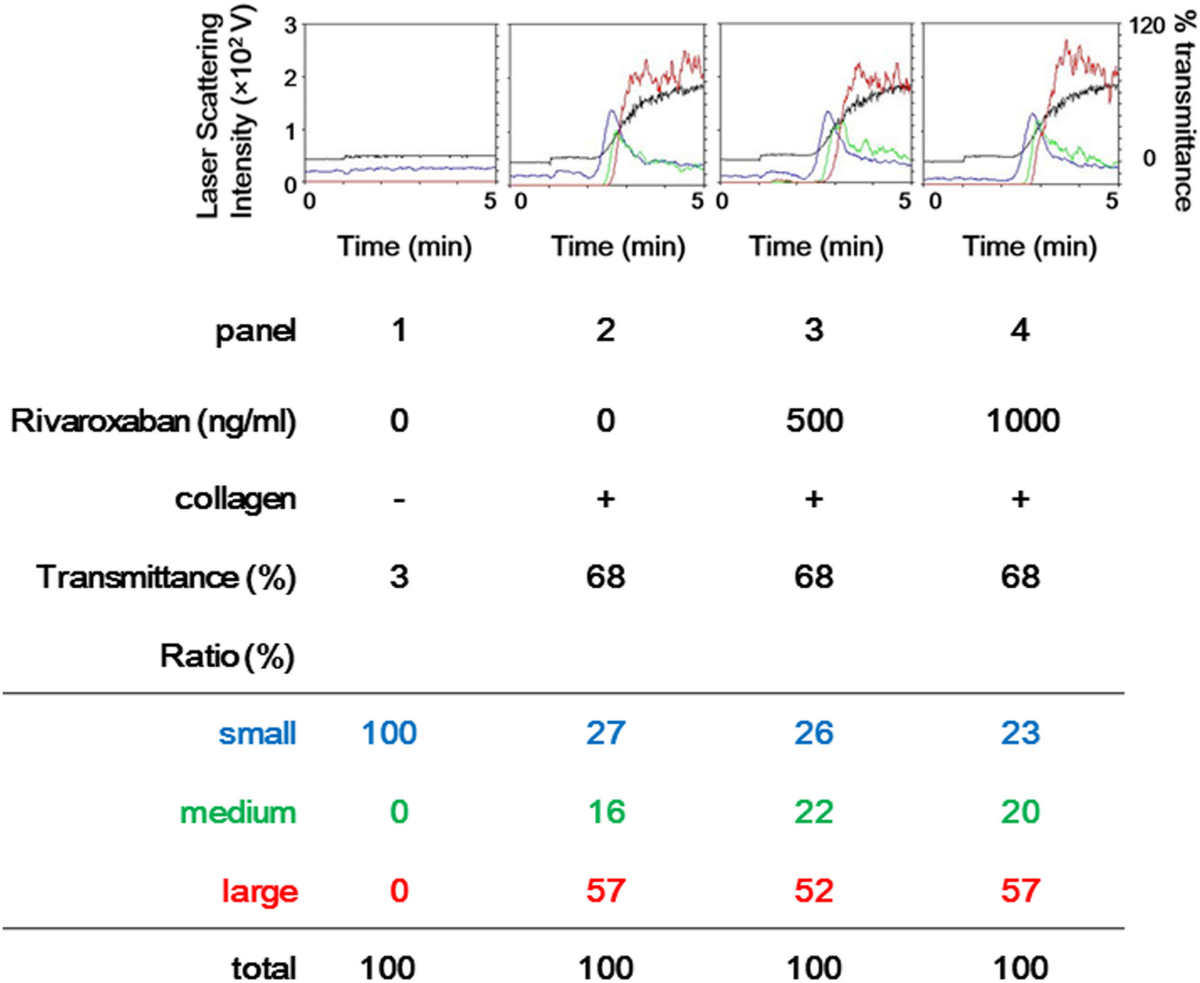
Representative data showing the effect of rivaroxaban on platelet aggregation induced by collagen. PRP was pretreated with various doses of rivaroxaban for 15 min, and then stimulated by 1.0 μg/ml collagen or vehicle for 5min. The reaction was terminated by the addition of ice-cold EDTA (10 mM) solution. Platelet aggregation was detected by an aggregometer with laser scattering system. The black line indicates the percentage of transmittance of each sample (PRP recorded as 0%, and PPP was recorded as 100%). The blue line indicates small aggregates (9–25 μm); the green line, medium aggregates (25–50 μm); the red line, large aggregates (50–70 μm). The distributions (%) of aggregated particle size were measured by AUC of each particle size. Representative results obtained from ten healthy donors are presented.

We first examined the effects of FXa inhibitors, rivaroxaban on platelet aggregation induced by 1.0 μg/ml of collagen. Rivaroxaban had little effect on the collagen-induced platelet aggregation in terms of maximum transmittance up to 1000 ng/ml (Fig 1). According to the analysis of the size of the aggregates, the distribution of aggregated particle sizes (small size, medium size, or large size) was not changed even when the platelets were treated with 1000 ng/ml of rivaroxaban.

### Effects of rivaroxaban or edoxaban on the collagen-induced phosphorylation of p44/p42 MAP kinase and HSP27 in human platelets

**Fig 2:**
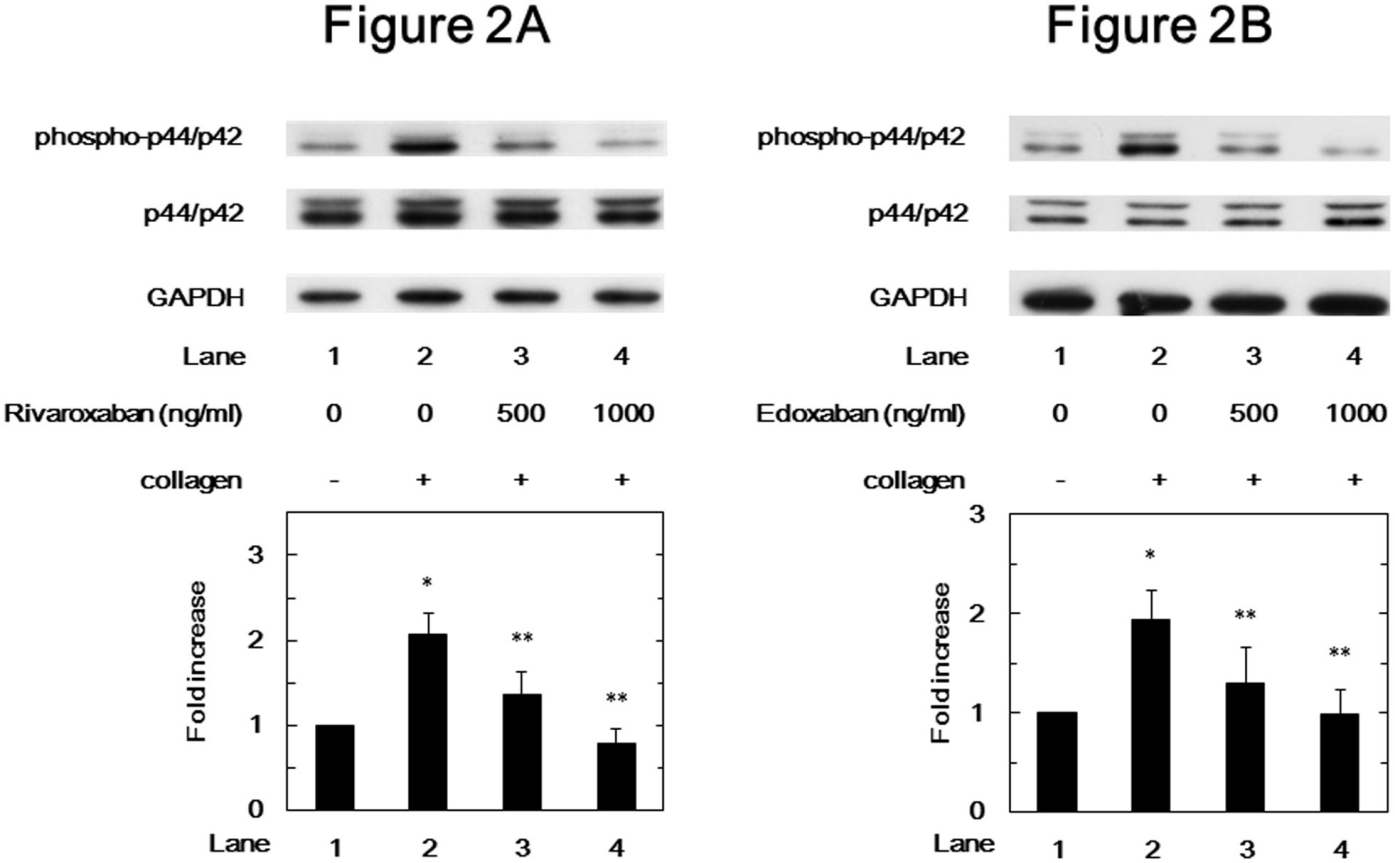
Effects of rivaroxaban and edoxaban on the collagen-induced phosphorylation of p44/p42 MAP kinase in human platelets. PRP was pretreated with various doses of rivaroxaban (A) or edoxaban (B) for 15 min, and then stimulated by 1.0 μg/ml collagen or vehicle for 5 min. The reaction was terminated by the addition of ice-cold EDTA (10 mM) solution. The lysate of platelets was subjected to Western blot analysis using antibodies against phospho-specific p44/p42 MAP kinase, p44/p42 MAP kinase or GAPDH. Representative results of rivaroxaban from ten healthy donors (A) and results of edoxaban from five healthy donors (B) are presented. The histogram shows quantitative representation of the collagen-induced levels obtained from laser densitometric analysis. The phosphorylation levels are expressed as the fold increase to the basal levels presented as lane 1. Each value represents the mean ± SEM. *p<0.05, compared to the value of the vehicle alone, and **p<0.05, compared to the value of collagen alone.

In order to investigate whether FXa inhibitor could affect the activation of p44/p42 MAP kinase induced by collagen or not, we examined the effects of FXa inhibitors on the collagen-induced phosphorylation of p44/p42 MAP kinase in human platelets. Rivaroxaban significantly suppressed the collagen-induced phosphorylation of p44/p42 MAP kinase in a dose-dependent manner in the range between 500 and 1000 ng/ml (Fig 2A). The inhibitory effect of rivaroxaban observed at 1000 ng/ml was approximately complete. In addition, edoxaban as well as rivaroxaban significantly attenuated the collagen-induced phosphorylation (Fig 2B).

**Fig 3:**
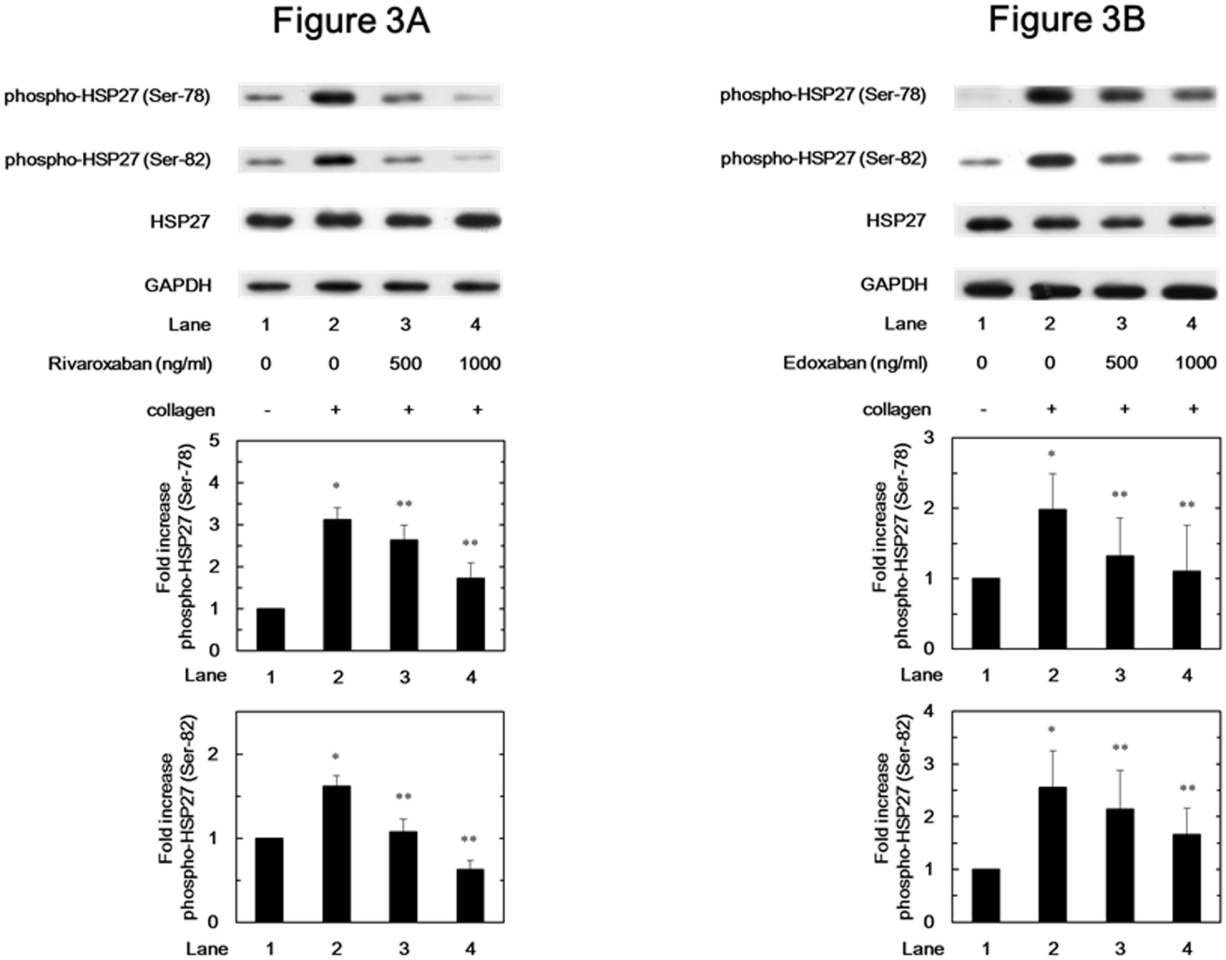
Effects of rivaroxaban and edoxaban on the collagen-induced phosphorylation of HSP27 in human platelets. PRP was pretreated with various doses of rivaroxaban (A) or edoxaban (B) for 15 min, and then stimulated by 1.0 μg/ml collagen or vehicle for 5 min. The reaction was terminated by the addition of ice-cold EDTA (10 mM) solution. The lysate of platelets was subjected to Western blot analysis using antibodies against phospho-specific HSP27 (Ser-78 and Ser-82), total HSP27 or GAPDH. Representative results of rivaroxaban from ten healthy donors (A) and results of edoxaban from five healthy donors (B) are presented. The histogram shows quantitative representation of the collagen-induced levels obtained from laser densitometric analysis. The phosphorylation levels are expressed as the fold increase to the basal levels presented as lane 1. Each value represents the mean ± SEM. *p<0.05, compared to the value of the vehicle alone, and **p<0.05, compared to the value of collagen alone.

We next examined the effects of FXa inhibitors on the phosphorylation of HSP27 induced by collagen in human platelets. Rivaroxaban significantly reduced the collagen-induced phosphorylation of HSP27 at ser-78 and ser-82 in a dose-dependent manner in the range between 500 and 1000 ng/ml (Fig 3A). Rivaroxaban caused 55% and complete reduction in the collagen-effect on the phosphorylation at Ser-78 and Ser-82, respectively. In addition, edoxaban significantly suppressed the collagen-induced HSP27 phosphorylation at Ser-78 and Ser-82 (Fig 3B).

### Effects of rivaroxaban or edoxaban on the collagen-induced phosphorylation of p38 MAP kinase in human platelets

**Fig 4:**
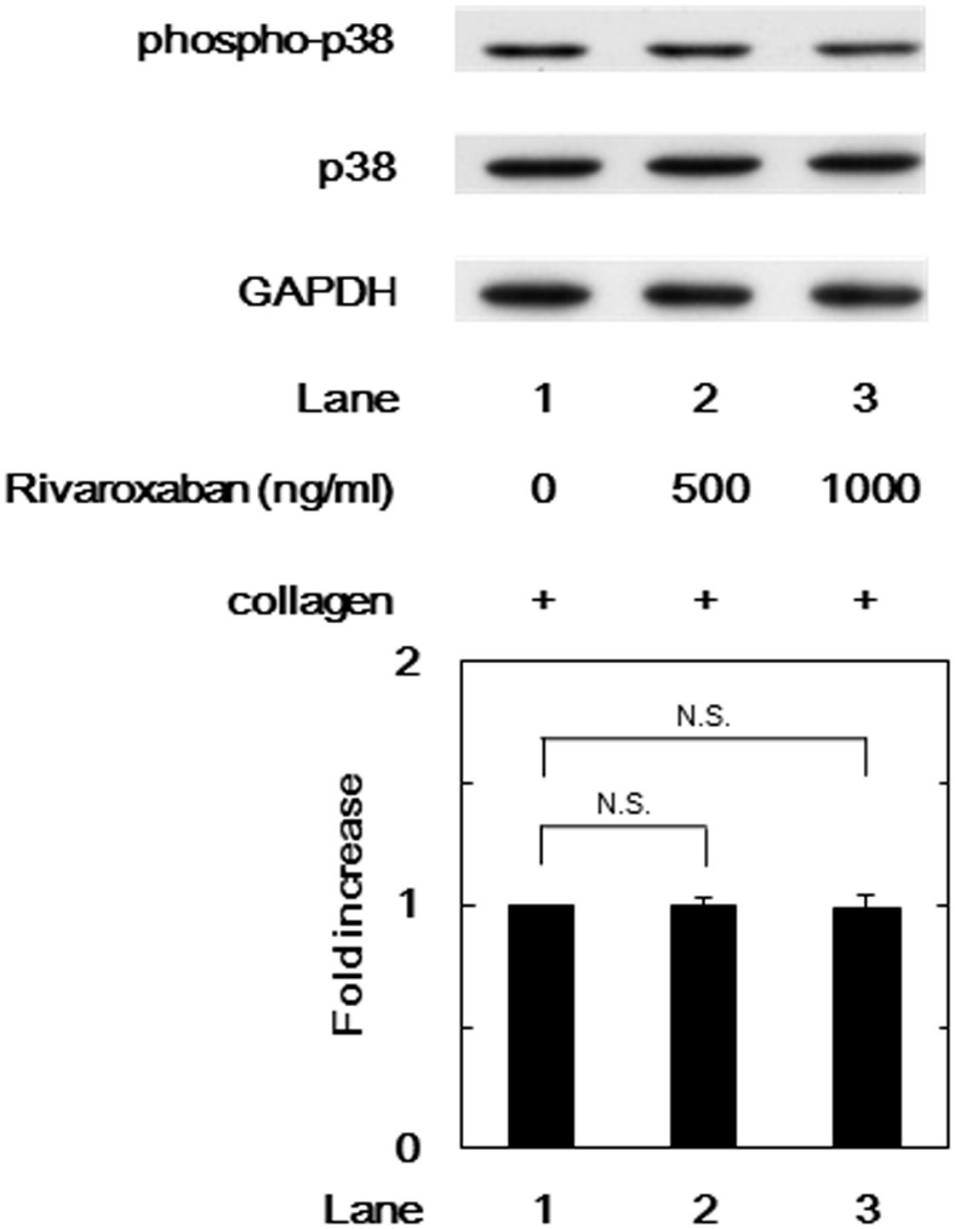
Effect of rivaroxaban on the collagen-induced phosphorylation of p38 MAP kinase in human platelets. PRP was pretreated with various doses of rivaroxaban for 15 min, and then stimulated by 1.0 μg/ml collagen for 5 min. The reaction was terminated by the addition of ice-cold EDTA (10 mM) solution. The lysate of platelets was subjected to Western blot analysis using antibodies against phospho-specific p38 MAP kinase, p38 MAP kinase or GAPDH. Representative results obtained from ten healthy donors are presented. The histogram shows quantitative representation of the collagen-induced levels obtained from laser densitometric analysis. The phosphorylation levels are expressed as the fold increase to the basal levels presented as lane 1. Each value represents the mean ± SEM. N.S. designates no significant difference between the indicated pairs.

In our previous study [28], we have demonstrated that the phosphorylation levels of collagen-induced p38 MAP kinase represent the hyperaggregability in platelets in type 2 DM patients. Thus, we examined the effects of FXa inhibitors on the collagen-induced phosphorylation of p38 MAP kinase. However, rivaroxaban hardly affected the phosphorylation of p38 MAP kinase induced by collagen in up to 1000 ng/ml (Fig 4).

### Effects of rivaroxaban or edoxaban on the collagen-induced secretion of PDGF-AB or the release of phosphorylated HSP27 from human platelets

**Fig 5:**
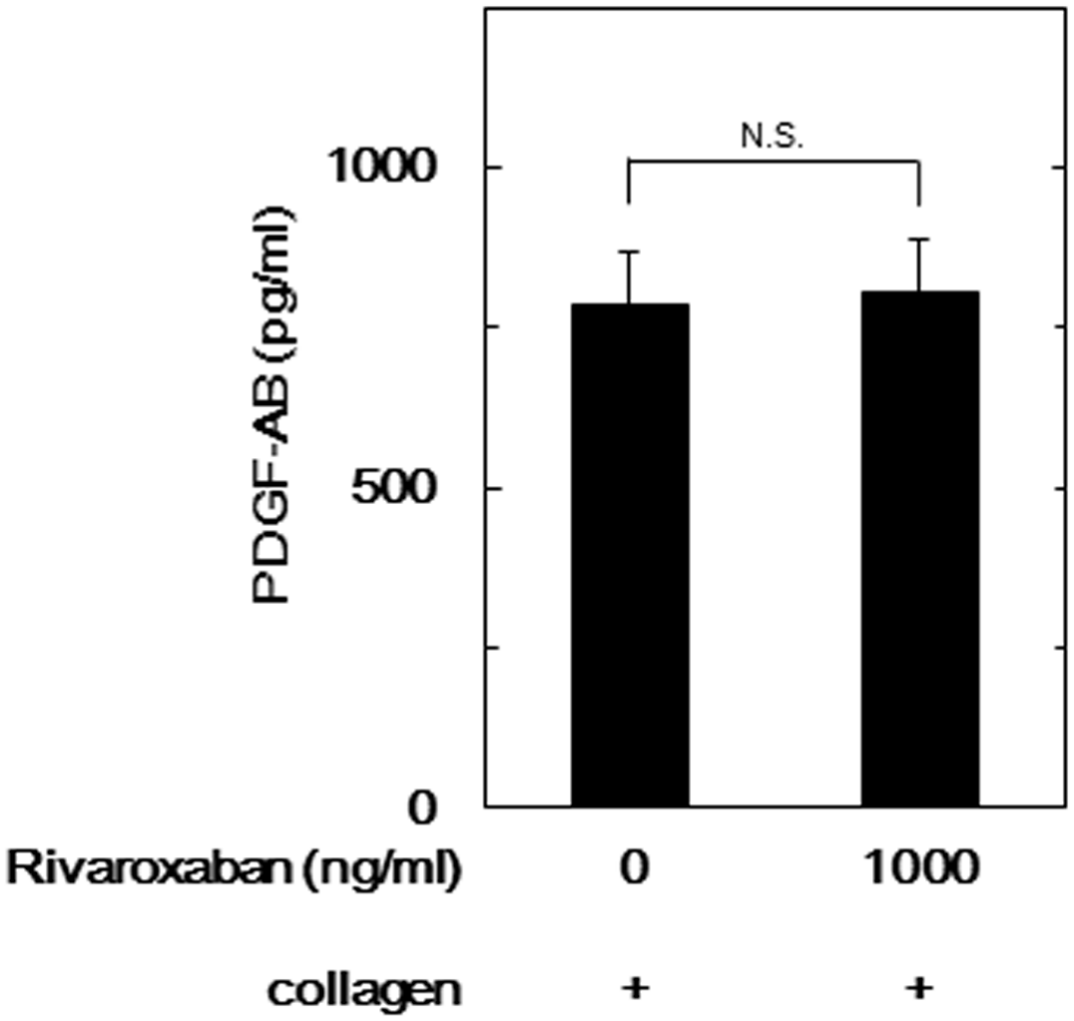
Effect of rivaroxaban on the collagen-stimulated PDGF-AB secretion from human platelets. PRP was pretreated with 1000 ng/ml of rivaroxaban or vehicle for 15 min, and then stimulated by 1.0 μg/ml collagen or vehicle for 5 min. The reaction was terminated by the addition of ice-cold EDTA (10 mM) solution. The conditional mixture was centrifuged at 10,000 x g at 4°C and the supernatants were then subjected to an ELISA for PDGF-AB. Values for unstimulated platelets have been subtracted from each data point. Results from ten healthy donors are shown. Each value represents the mean ± SEM. N.S. designates no significant difference between the indicated pairs.

We examined the effects of FXa inhibitors on the PDGF-AB secretion induced by collagen. The secretion of PDGF-AB from collagen-stimulated platelets was not affected by 1000 ng/ml of rivaroxaban compared to collagen alone (Fig 5).

**Fig 6:**
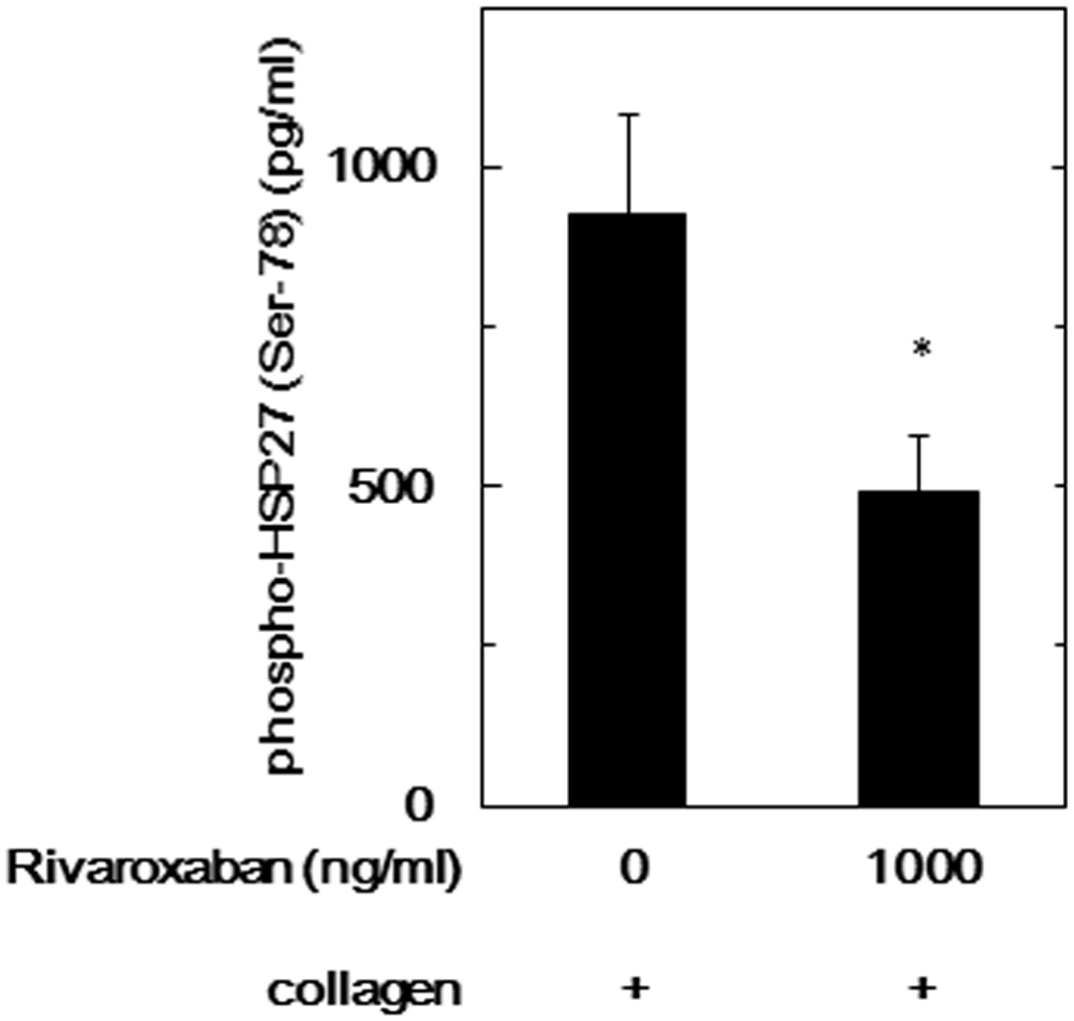
Effect of rivaroxaban on the collagen-stimulated HSP27 release from human platelets. PRP was pretreated with 1000 ng/ml of rivaroxaban or vehicle for 15 min, and then stimulated by 1.0 μg/ml collagen or vehicle for 5 min. The reaction was terminated by the addition of ice-cold EDTA (10 mM) solution. The conditional mixture was centrifuged at 10,000 x g at 4°C and the supernatants were then subjected to an ELISA for phosphorylated HSP27. Values for unstimulated platelets have been subtracted from each data point. Results from ten healthy donors are shown. Each value represents the mean ± SEM. *p<0.05, compared to the value of the collagen alone.

We have recently reported that HSP27 is released from human platelets accompanied with the collagen-stimulated phosphorylation of HSP27 [25]. Therefore, we next examined the effects of FXa inhibitors on the collagen-induced release of phosphorylated HSP27 from human platelets. The release of phosphorylated HSP27 stimulated by collagen was significantly suppressed by 1000 ng/ml of rivaroxaban compared to collagen alone (Fig 6).

### Effect of collagen on HSP27 phosphorylation (Ser-78) in platelets in patients after rivaroxaban administration

**Fig 7:**
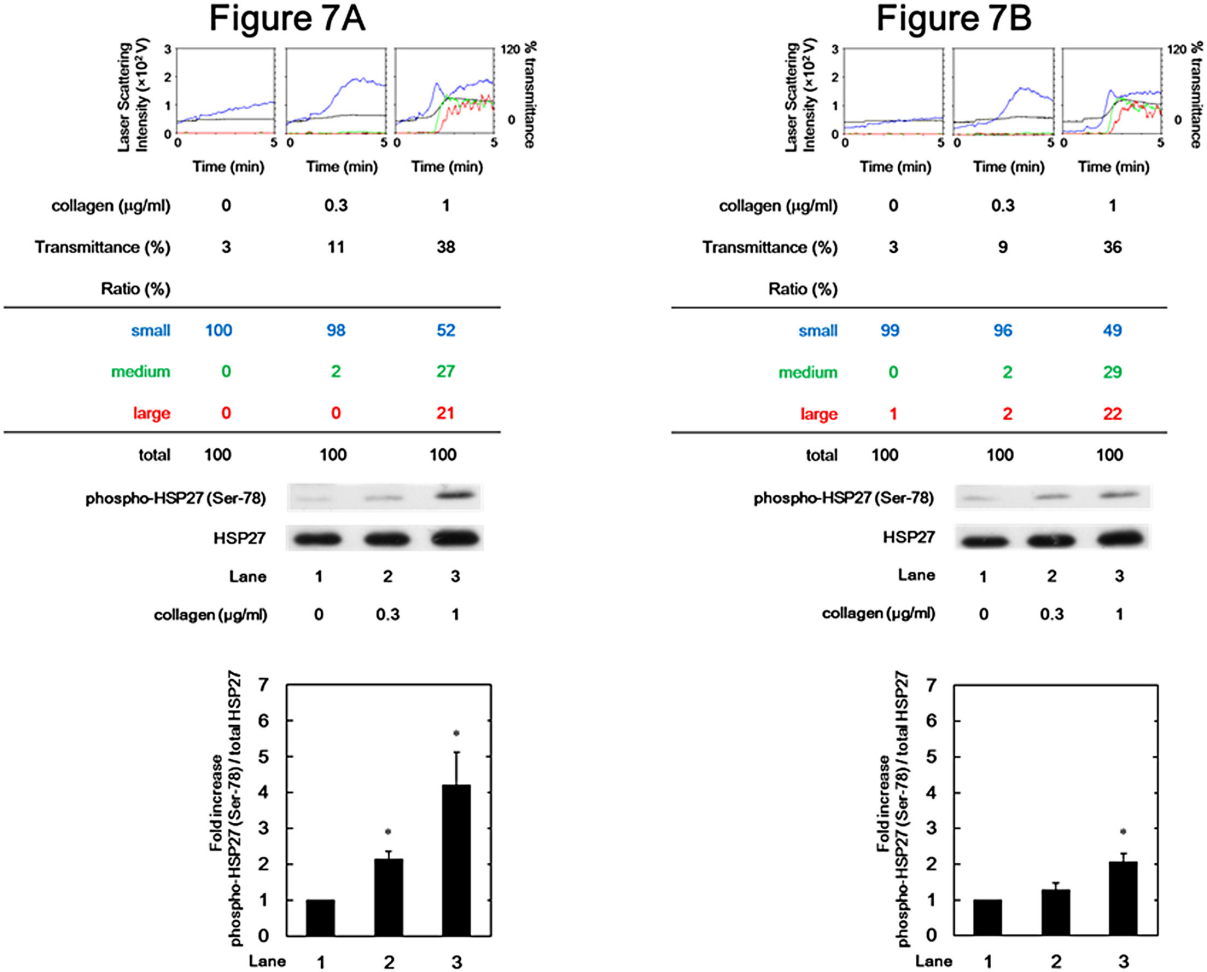
Representative data showing the effect of collagen on platelet aggregation and HSP27 phosphorylation (Ser-78) in platelets in patients after rivaroxaban administration. PRP was stimulated by various doses of collagen for 5 min, and the reaction was terminated by the addition of ice-cold EDTA (10 mM) solution. The black line indicates the percentage of transmittance of each sample (PRP recorded as 0%, and PPP was recorded as 100%). The blue line indicates small aggregates (9–25 μm); the green line, medium aggregates (25–50 μm); the red line, large aggregates (50–70 μm). The distributions (%) of aggregated particle size were measured by AUC of each particle size. The lysates of platelets were subjected to Western blot analysis using antibodies against HSP27, or phospho-specific HSP27 (Ser-78). Representative results obtained from five patients before rivaroxaban administration (A) and after the administration for 2 days (B). In the lower panel, the histogram shows quantitative representation of the collagen-induced phosphorylation levels obtained from laser densitometric analysis. The levels are expressed as the fold increase to the basal levels presented as lane 1. Each value represents the mean ± SEM. *p<0.05, compared to the value of the vehicle alone.

We further examined the effect of collagen on HSP27 phosphorylation (Ser-78) using platelets from non-valvular atrial fibrillation or deep venous thrombosis patients after rivaroxaban administration. Representative result before (Fig 7A) and 2-days-after (Fig 7B) administration of rivaroxaban is shown. According to the analysis of an aggregometer with laser scattering system, rivaroxaban hardly affected the platelet aggregation induced by collagen in terms of both maximum transmittance and the ratio of the size of platelet aggregates compared to before rivaroxaban administration. On the other hand, administration of rivaroxaban markedly suppressed the collagen-induced phosphorylation of HSP27 (Ser-78) (Fig 7A and 7B).

## Discussion

In the present study, we investigated the direct effects of FXa inhibitors such as rivaroxaban and edoxaban on collagen-induced activation of human platelets. It has previously been reported that FXa inhibitors have no effect on platelet aggregation induced by several agonists such as collagen, ADP, ristocetin or TX agonist using light transmittance aggregometry [12,13,29]. It is currently recognized that FXa inhibitors do not directly affect platelet aggregation. As for the effects of anticoagulant on platelet aggregation, we have previously reported that AT-III reduces the collagen-induced platelet aggregation using aggregometer with laser scattering system, which can present the size of platelet aggregates in addition to transmittance [14]. We first examined the effect of FXa inhibitor on human platelets using aggregometer with the system. We found that rivaroxaban truly did not inhibit the collagen-induced platelet aggregation in terms of the distribution of platelet aggregate sizes as well as transmittance *in vitro*. In addition, we examined the effect of collagen on platelet aggregation in patients with non-valvular atrial fibrillation or deep venous thrombosis before and after rivaroxaban administration, and showed that rivaroxaban had little effect on platelet aggregation using laser scattering system. Based on our findings, which is generally consistent with the previous reports [12,13], it is most likely that FXa inhibitor failed to affect collagen-induced human platelet aggregation.

We have previously reported that the collagen-induced phosphorylation of HSP27 is positively regulated by the activation of Rac-dependent p44/p42 MAP kinase in human platelets [23,24]. Therefore, we next investigated the effects of FXa inhibitors on the collagen-stimulated phosphorylation of HSP27 and p44/p42 MAP kinase in human platelets *in vitro*. We demonstrated that rivaroxaban and edoxaban significantly attenuated the phosphorylation levels of p44/p42 MAP kinase and those of HSP27 at two serine residues, Ser-78 and Ser-82. Additionally, we examined the effect of collagen on the phosphorylation of HSP27 at Ser-78 in patients before and after rivaroxaban administration, and found that the administration of rivaroxaban for 2 days remarkably attenuated the phosphorylation of HSP27 (Ser-78) in the collagen-stimulated platelets. It has been shown that the phosphorylation of p38 MAP kinase is correlated with the phosphorylation of HSP27 [22]. We have previously reported that collagen-induced phosphorylation levels of p38 MAP kinase reflects platelet aggregability in type 2 DM patients [28]. In the present study, however, FXa inhibitors did not suppress the phosphorylation levels of p38 MAP kinase induced by collagen in human platelets. Taking our findings into account, it is probable that FXa inhibitor selectively attenuates the collagen-induced HSP27 phosphorylation via p44/p42 MAP kinase but not the p38 MAP kinase pathway in human platelets.

The phosphorylation of HSP27 is reportedly associated with the cytoskeleton and actin polymerization in activated human platelets [30,31]. We next investigated the relationship between FXa inhibitor and granule secretion such as PDGF-AB from human platelets. We demonstrated that collagen-induced secretion of PDGF-AB was not affected even by the 1000 ng/ml of rivaroxaban compared to collagen alone. In our recent study [25], we have shown that phosphorylated HSP27 is released from human platelets accompanied with its phosphorylation induced by collagen. Therefore, we examined the effects of FXa inhibitors on the release of phosphorylated HSP27 from collagen-stimulated platelets. We showed that 1000 ng/ml of rivaroxaban significantly suppressed the collagen-induced release of phosphorylated HSP27 from human platelets. It is firmly established that HSP27 exists in an unphosphorylated aggregated form under unstimulated condition, and HSP27 is dissociated in association with phosphorylation, resulting into dissociated form, dimer or monomer [15,21]. It seems likely that the decreased size of HSP27 accompanied with phosphorylation is released from human platelets. Based on our findings, it is most likely that FXa inhibitor reduces the release of phosphorylated HSP27 from human platelets stimulated by collagen due to the inhibition of HSP27 phosphorylation via p44/p42 MAP kinase.

Accumulating evidence indicates that HSP27 possess extracellular effects besides intracellularly functions as a molecular chaperone [16,32]. HSP27 released from macrophage reportedly has an anti-atherosclerosis effect [33]. On the other hand, it has been shown that HSP27 upregulates not only anti-inflammatory factors such as interleukin (IL)-10 but also pro-inflammatory factors including IL-1β via the activation of NF-κB in macrophages [34]. Recently, it has been reported that human myocardium after global ischemia releases HSP27, eventually resulting in exhibiting the pro-inflammatory effect through toll-like receptors in mouse coronary vascular endothelial cells [35]. On the other hand, thrombus formation originates from the initial tethering of platelets at the sites of exposed subendothelial collagen [1,2]. We have recently reported that collagen-activated human platelets release HSP27 accompanied with its phosphorylation [25]. Therefore, human platelets could be a source of extracellular HSP27 at the site of damaged vessel walls. Based on our present findings, it is possible that FXa inhibitor has an anti-inflammatory effect for vascular endothelial cells via reduction of the release of HSP27 from collagen-stimulated platelets at the sites of vascular injury. FXa is a feasible target for anticoagulation [36], but have also been shown to possess other biological and pathophysiological effect through PAR [37–39]. The direct cellular effects of FXa are reportedly responsible for promoting inflammation and atherosclerosis [37,39]. Since the effects of FXa on human platelets might be either directly via binding and activation of PAR or indirectly through the generation of thrombin, FXa inhibitor could affect these two pathways. Rivaroxaban has been approved in Europe for not only the treatment of symptomatic deep vein thrombosis [40] or the prevention ischemic stroke due to non-valvular atrial fibrillation [41], but also for the additional treatment on antiplatelet therapy in patients with coronary artery disease [42,43]. Therefore, besides anticoagulant effect of rivaroxaban, anti-inflammatory effect for coronary vascular endothelium via suppression of HSP27 release from human platelets might contribute to a reduction in risk for myocardial death and stent thrombosis in acute coronary syndrome patients. Further investigations are necessary to elucidate the exact role of FXa inhibitors in human platelets

In conclusion, our present findings strongly suggest that FXa inhibitor suppresses the collagen-stimulated phosphorylation of HSP27 via p44/p42 MAP kinase in human platelets, eventually resulting in the inhibition of the release of phosphorylated HSP27 from human platelets.
